# Mismatch negativity and neural adaptation: Two sides of the same coin. Response: Commentary: Visual mismatch negativity: a predictive coding view

**DOI:** 10.3389/fnhum.2016.00013

**Published:** 2016-01-29

**Authors:** Gábor Stefanics, Jan Kremláček, István Czigler

**Affiliations:** ^1^Translational Neuromodeling Unit, Institute for Biomedical Engineering, University of Zurich and ETH ZurichZurich, Switzerland; ^2^Laboratory for Social and Neural Systems Research, Department of Economics, University of ZurichZurich, Switzerland; ^3^Faculty of Medicine in Hradec Králové, Department of Pathological Physiology, Charles University in PragueHradec Králové, Czech Republic; ^4^Research Center for Natural Sciences, Institute of Cognitive Neuroscience and Psychology, Hungarian Academy of SciencesBudapest, Hungary

**Keywords:** mismatch negativity (MMN, vMMN), stimulus-specific adaptation, refractoriness, repetition suppression, habituation, modeling

Our recent paper (Stefanics et al., 2014) provided a comprehensive review of the visual MMN literature from a predictive coding perspective. We argued the MMN reflects a phenomenon consisting of multiple neural processes underlying the initial response to rare, unpredicted stimuli and the attenuation of this response over subsequent stimulus repetitions. We think repetition suppression (RS) is an important process of the compound mismatch phenomenon. In our review we often referred to the contribution of the repetition effect to the MMN as “refractoriness” and highlighted that predictive coding offers a unified framework to explain the multiple mismatch processes.

O'Shea (2015) argued that a “better term for refractoriness is ‘adaptation’ [and that] adaptation ought to be harmonized into any complete MMN explanation.” O'Shea concluded that “replacing ‘refractoriness’ in the MMN vocabulary with adaptation terms and searching for a rapprochement between adaptation and MMN could bring considerable explanatory benefits.”

The term “refractoriness” was originally used in the MMN field to describe response attenuation for repeated events, linked to sensory memory formation (Näätänen and Picton, 1987). The deviant-minus-standard difference caused by repetition was attributed to neuronal fatigue, as opposed to the difference caused by genuine mismatch-related responses. The MMN community considered the standard-related effects irrelevant to deviance detection. In other fields which focus on stimulus-specific adaptation (SSA) instead of deviance detection (psychophysics, cellular electrophysiology, and neuroimaging) RS is attributed to active memory processes. Thus, there are important differences in where the emphasis of RS-related research lies in the MMN and other fields.

We agree with O'Shea (2015) that harmonizing adaptation into any theoretical treatment of the MMN is necessary and beneficial. In fact, we aimed to contribute to the harmonization process by discussing not only MMN but also adaptation in our review. Replacing refractoriness in the MMN vocabulary with adaptation terms would help the field acknowledge that deviance detection is intricately linked to the process of regularity extraction, which in turn is linked to adaptation or RS. Nevertheless, each of these terms is used to describe several related concepts and phenomena, and it is hard to pin one concept on one term.

In the 1980s it was common to refer to repetition effects for ERPs as refractoriness. Using this term to describe changes in scalp-recorded ERPs was perhaps not the best choice for the MMN field, because it emphasizes the passive nature of the response attenuation at the single neuron level whereas several line of evidence suggests that RS in not the result of refractory-like fatigue. However, simply replacing refractoriness in the MMN vocabulary with adaptation terms might create the false impression that network mechanisms underlying RS (Ibbotson, 2005; Grill-Spector et al., 2006) are well understood. This should be avoided, therefore harmonizing adaptation and MMN should be done with caution.

RS is a ubiquitous phenomenon, observed in countless experiments in several distinct fields. However, integrating results from different fields using disparate methodologies is not straightforward. For example, several attempts have been made to identify the single-cell correlates of scalp-recorded MMN. Auditory SSA is associated with midlatency potentials and is the closest known single-neuron phenomenon of MMN (Escera and Malmierca, 2014; Nelken, 2014). The magnitudes of SSA and MMN are both negatively correlated with the probability of the deviant but positively correlated with the difference between standard and deviant. However, an important difference is the earlier timing of SSA relative to MMN, which led Nelken and Ulanovsky (2007) to suggest that SSA is a correlate of change detection in the primary auditory cortex upstream of MMN, and that MMN itself is a compound response of primary and higher-level cortical areas with longer response latencies.

SSA is present at nearly all stages in visual processing (Solomon and Kohn, 2014) and involves at least three mechanisms, including (1) somatic afterhyperpolarization, (2) synaptic depression due to the depletion of vesicles from the presynaptic terminal, and (3) synaptic (network) mechanisms (Kohn, 2007). Because the refractory state of a neuron after spiking is too short to be responsible for the ERP amplitude decrease after repeated stimulation and synaptic depletion also occurs only at higher stimulation rates than in MMN experiments, RS in MMN experiments likely results from network mechanisms which are not fully understood yet in the visual system.

Results of visual ERP studies of adaptation have been variable. Several studies reported attenuation of some ERP components (Schweinberger et al., 2004; Fiebach et al., 2005; Kovács et al., 2006; Harris and Nakayama, 2007; Huber et al., 2008; Caharel et al., 2009; Vizioli et al., 2010; Vakli et al., 2014). However, some of the above and other studies (Puce et al., 1999; Andrade et al., 2015) also observed repetition enhancement, or no change. Thus, ERP correlates of visual adaptation warrants further investigation.

Attempts to disentangle different processes underlying RS and change detection has led the MMN field to come up with smart experimental paradigms, such as the equiprobable control, which allows studying effects of stimulus repetition and change separately (Schröger and Wolff, 1996; Ruhnau et al., 2012). Although experimental manipulations indeed help disentangle compound processes, a principled approach might be using computational models (May and Tiitinen, 2010; Garagnani and Pulvermüller, 2011; Wacongne et al., 2012). Dynamic causal modeling (DCM) has been successfully used to compare large-scale network models of MMN (Kiebel et al., 2007; Garrido et al., 2008, 2009) which incorporate hypotheses of both adaptation and change detection. Further recent modeling studies demonstrate the potential of predictive coding to provide a comprehensive explanation of MMN phenomenology (Lieder et al., 2013a). Results of Lieder et al. (2013b) suggest that the MMN reflects approximate Bayesian learning, and that the MMN-generating process adjusts a probabilistic model of the environment using prediction errors.

## Conclusion

Using neurobiologically informed modeling frameworks which rely on Bayesian probability theory might provide rapprochement between adaptation and MMN. By focusing on computational mechanisms (Marr, 1982) instead of phenomenological description of neural responses, such an approach might lead to the emergence of a vocabulary that is abstract enough to support communication across diverse research fields which nevertheless study similar phenomena.

## Author contributions

GS, JK, and IC wrote the paper.

### Conflict of interest statement

The authors declare that the research was conducted in the absence of any commercial or financial relationships that could be construed as a potential conflict of interest.
